# Supplementary material for: HiIDDD: a high-throughput imaging pipeline for the quantitative detection of DNA damage in primary human immune cells
Source: Sci Rep. 2022 Apr 15;12:6335. doi: 10.1038/s41598-022-10018-0 (PMC9022135; doi:10.1038/s41598-022-10018-0)
Supplement: Supplementary file 1 — Supplementary Information. [file 41598_2022_10018_MOESM1_ESM.pdf]

**a**

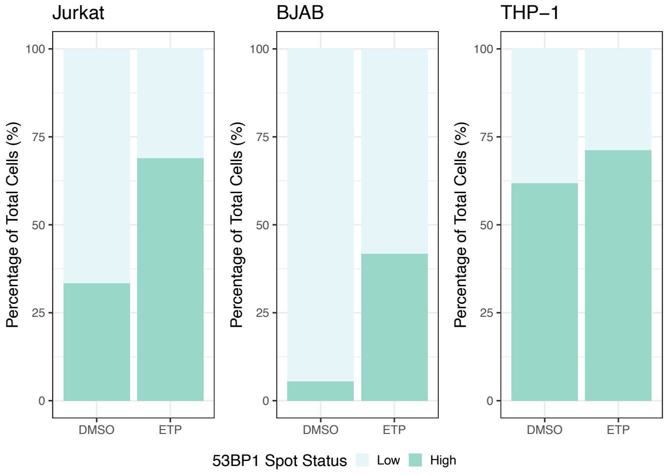


**b**

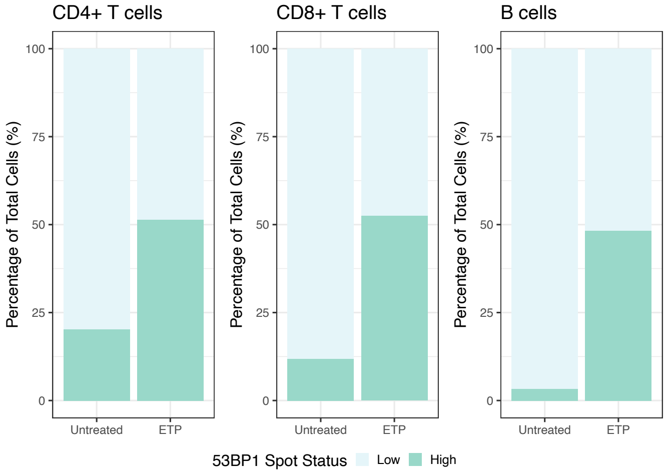


**c**

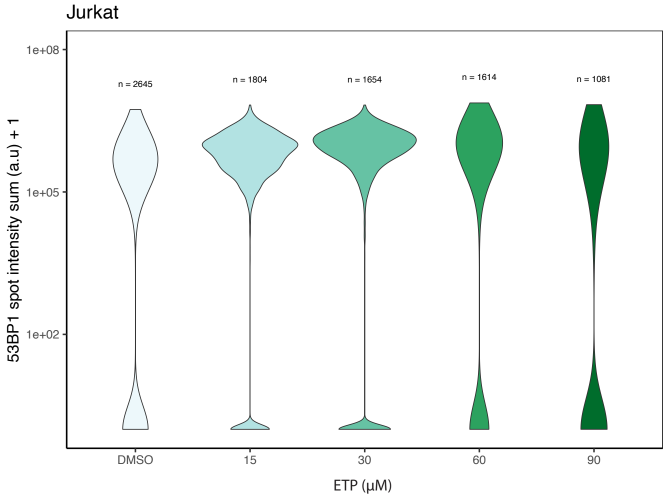


**d**

| ETP treatment (μM) | Mean (a.u.) | SD (a.u.) | 53BP1 Positive (%) |
|---|---|---|---|
| DMSO | 4.67E+05 | 5.86E+05 | 33.3 |
| 15 | 8.65E+05 | 8.32E+05 | 58 |
| 30 | 1.07E+06 | 9.34E+05 | 69 |
| 60 | 9.70E+05 | 1.00E+06 | 59.2 |
| 90 | 6.64E+05 | 9.35E+05 | 39.5 |

**e**

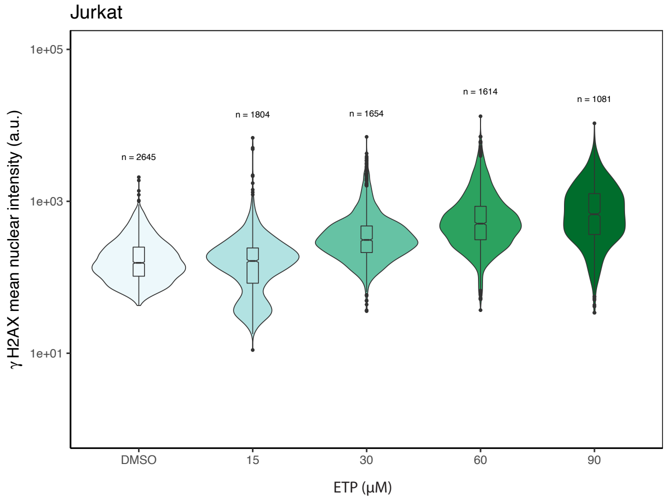


**f**

| ETP treatment (μM) | Mean (a.u.) | SD (a.u.) | $\gamma$-H2AX Positive (%) |
|---|---|---|---|
| DMSO | 2.00E+02 | 1.51E+02 | 12.4 |
| 15 | 2.02E+02 | 3.05E+02 | 10.6 |
| 30 | 4.32E+02 | 4.60E+02 | 42.3 |
| 60 | 7.73E+02 | 8.64E+02 | 70.4 |
| 90 | 9.52E+02 | 9.02E+02 | 76.4 |

**Supplementary Fig. 1) Detection of 53BP1 spot intensity in immune cells.** 53BP1 positive cells were characterized in each treatment group for **a)** immune cell lines (Jurkat, BJAB, and THP-1) and **b)** $CD4^{+}$T cells, CD8+T cells, monocytes, and B cells. The same samples as in figures 2 and 3 are used. ETP Dose-response assay (0-90 µM) in Jurkat cell line for **c, d)** 53BP1 spot intensity and **e, f)** $\gamma$-H2AX mean nuclear intensity detection.

**a**

| # | Donor | Age | Sex |
|---|---|---|---|
| 1 | ND791 | 30 | F |
| 2 | ND782 | 31 | F |
| 3 | Gt016 | 42 | F |
| 4 | Gt024 | 47 | M |
| 5 | Gt030 | 49 | M |
| 6 | ND550 | 54 | F |
| 7 | ND797 | 58 | F |
| 8 | Gt004 | 62 | M |
| 9 | ND334 | 67 | F |
| 10 | Gt029 | 85 | M |

**Supplementary Fig. 2) List of donors used in batch processing assay. a)** For each listed donor in Figure 5, donors were identified by age and sex.
